# Kynurenine 3-Monooxygenase Gene Associated With Nicotine Initiation and Addiction: Analysis of Novel Regulatory Features at 5′ and 3′-Regions

**DOI:** 10.3389/fgene.2018.00198

**Published:** 2018-06-13

**Authors:** Hassan A. Aziz, Abdel-Salam G. Abdel-Salam, Mohammed A. I. Al-Obaide, Hytham W. Alobydi, Saif Al-Humaish

**Affiliations:** ^1^College of Arts and Sciences, Qatar University, Doha, Qatar; ^2^School of Medicine, Texas Tech University Health Sciences Center, Amarillo, TX, United States; ^3^Biomedica, LLC, Sterling Heights, MI, United States

**Keywords:** *KMO*, kynurenine 3-monooxygenase, gene expression, alternative promoters, non-coding RNA, nicotine, α7nAChR, kynurenic acid

## Abstract

Tobacco smoking is widespread behavior in Qatar and worldwide and is considered one of the major preventable causes of ill health and death. Nicotine is part of tobacco smoke that causes numerous health risks and is incredibly addictive; it binds to the α7 nicotinic acetylcholine receptor (α7nAChR) in the brain. Recent studies showed α7nAChR involvement in the initiation and addiction of smoking. Kynurenic acid (KA), a significant tryptophan metabolite, is an antagonist of α7nAChR. Inhibition of kynurenine 3-monooxygenase enzyme encoded by *KMO* enhances the KA levels. Modulating *KMO* gene expression could be a useful tactic for the treatment of tobacco initiation and dependence. Since *KMO* regulation is still poorly understood, we aimed to investigate the 5′ and 3′-regulatory factors of *KMO* gene to advance our knowledge to modulate *KMO* gene expression. In this study, bioinformatics methods were used to identify the regulatory sequences associated with expression of *KMO*. The displayed differential expression of *KMO* mRNA in the same tissue and different tissues suggested the specific usage of the *KMO* multiple alternative promoters. Eleven *KMO* alternative promoters identified at 5′-regulatory region contain TATA-Box, lack CpG Island (CGI) and showed dinucleotide base-stacking energy values specific to transcription factor binding sites (TFBSs). The structural features of regulatory sequences can influence the transcription process and cell type-specific expression. The uncharacterized LOC105373233 locus coding for non-coding RNA (ncRNA) located on the reverse strand in a convergent manner at the 3′-side of *KMO* locus. The two genes likely expressed by a promoter that lacks TATA-Box harbor CGI and two TFBSs linked to the bidirectional transcription, the NRF1, and ZNF14 motifs. We identified two types of microRNA (miR) in the uncharacterized LOC105373233 ncRNA, which are like hsa-miR-5096 and hsa-miR-1285-3p and can target the miR recognition element (MRE) in the *KMO* mRNA. Pairwise sequence alignment identified 52 nucleotides sequence hosting MRE in the *KMO* 3′ UTR untranslated region complementary to the ncRNA LOC105373233 sequence. We speculate that the identified miRs can modulate the *KMO* expression and together with alternative promoters at the 5′-regulatory region of *KMO* might contribute to the development of novel diagnostic and therapeutic algorithm for tobacco smoking.

## Introduction

Morbidity and mortality caused by smoking behavior are avoidable. Bioinformatics and genomics studies can aid in tobacco cessation and treatment. The targets for nicotine are the nicotinic acetylcholine receptors (nAChRs; Mineur and Picciotto, 2008). Inherited genetic variations in nAChRs, α, and β nAChRs could predict response to smoking cessation therapy (Wassenaar et al., 2011; Broms et al., 2012; Zhu et al., 2014). Variants of α and β nAChRs genes involved in the behavioral and cognitive performance identify patients’ benefit from personalized smoking cessation intervention and as markers for lung cancer diagnosis (Chen and Bierut, 2013; Chen et al., 2015; Pérez-Rubio et al., 2016; Chen L.S. et al., 2018). Furthermore, variants of the *CHRNA7* locus encoding the α7nAChR are linked to smoking behavior and engaged in the initiation of smoking in healthy individuals (Brunzell et al., 2015).

The liability to smoking initiation, nicotine dependence, and intervention encouraged us to study the unexplored genomic and epigenomic regulatory factors of kynurenine 3-monooxygenase gene, *KMO*. The gene linked to several neuropsychiatric diseases by modulating KA cellular levels (Erhardt et al., 2017). The *KMO* gene involvement in neurological disorders identified in Huntington disease and schizophrenia (Amaral et al., 2013). Also, it is worth mentioning that the α7nAChRs notably associated with smoking habit in individuals with schizophrenia (Brunzell et al., 2015), prepulse inhibition of startle, and cognitive function, which can be enhanced by nicotine (Pinnock et al., 2015). Besides the importance of *KMO* in clinical research of neurological ailments, there is a potential for using the *KMO* gene for treating tobacco addiction and cessation. The kynurenine 3-monooxygenase enzyme has a significant role in tryptophan metabolism, kynurenine pathway, and the availability of kynurenic acid (KA) (Albuquerque and Schwarcz, 2013). During past two decades, a series of studies showed Ro 61-8048, kynurenine 3-monooxygenase inhibitor, a potential therapeutic target for neurodegenerative and neurologic disorders and promoted increased levels of the KA in the brain that reduced the cannabinoid abuse and prevented relapse (Röver et al., 1997; Justinova et al., 2013; Secci et al., 2017; Gao et al., 2018). Intriguingly, the KA is an antagonist of α7 nicotinic acetylcholine receptors (α7nAChRs; Justinova et al., 2013). Even though a recent study showed that kynurenine 3-monooxygenase inhibition by Ro 61-8048 is promising for the treatment of nicotine addiction (Secci et al., 2017), there could also be genomic approaches for this problem. There are no data on the precise mode of regulatory factors associated with modulation of *KMO* expression and their potential use in the treatment of tobacco initiation and addiction. Thus, it is vital to gain a better understanding of the *KMO* genomic regulatory components associated with the control of the expression. We noticed several promoters in the databases for *KMO* gene, but no information is available on the regulatory elements involved in the *KMO* expression. For example, transcription factor binding sites (TFBSs) consensus motifs, CpG Islands (CGIs), and the structural features of the *KMO* alternative promoters. Also, we speculated the potential involvement of the non-coding RNA (ncRNA) LOC105373233 locus in the regulation of *KMO* expression. LOC105373233 locus is the convergent pair of the *KMO* gene. Better knowledge of the genomic and epigenomic features of *KMO* can promote novel approaches to modulate *KMO* expression by genome editing methods. Such approaches could be utilized to modulate KA levels and can be used as biomarkers and targets for tobacco smoking cessation therapy.

In this study, we aimed to investigate the *KMO* regulatory factors to advance our knowledge to control the *KMO* gene expression. We investigated the motifs of the TFBSs and CGIs in the *KMO* alternative promoters. Also, we investigated the structural features of *KMO* regulatory sequences analyzed by dinucleotide stacking energy, and potential influence of the ncRNA uncharacterized LOC105373233 locus mapped at *KMO* 3′-region on the expression of *KMO* gene.

## Materials and Methods

### Genomic Context of *KMO* Regulatory Region

The alternative promoters and genomic features of *KMO* gene searched using genomics databases shown in Supplementary Table S1. The tools in the databases used to analyze the regulatory sequences. Identifying the local similarities between two sequences analyzed by a rigorous algorithm based on the LALIGN application of the EMBOSS Matcher tool. Data from biogps.org used to vindicate our hypothesized approach that variations in the human *KMO* gene expression in various tissues take advantage of specific regulatory elements located in the alternative promoters of *KMO* gene. Expression values from Affymetrix chips relate to fluorescence intensity; the biogps.org uses gcrma (Wu et al., 2013, 2016). The biogps.org database gives the minimum of two measurements for each tissue. Neural Network Promoter Prediction (Reese, 2001) and Promoter2.0 Prediction Server (Knudsen, 1999) used to predict *KMO* promoters at the 5′-side of the *KMO* gene. The map sites of the *KMO* alternative promoters and other regulatory sequences updated to hg38 version of human genome sequence by using the BLAT tool of UCSC Genome Browser. The 5′ and 3′ UTR regions of the *KMO* mRNA analyzed by the UCSC Genome Browser sequence tools.

### Analysis of Functional and Structural Regulatory Features of *KMO* Promoters

The motifs of 20 TFBSs, namely: TATA-box (TATAWA), INR (YYANWYY), DTIE (GBBRDNHGG), BRE (SSRCGCC), DPE (RGWCGTG), M3 (SCGGAAGY), M22 (TGCGCANK), HIF (RCGTG), EBF3 (CCCNNGGG), Sox (WWCAAWG), P53 (RRRCWWGY), Oct4 (TTTKSWTW), PU.1 (GAGGAA), Nanog (SRSSATTANS), c-myc (CACRTG), MAD (CCGNCGCG), GAF (GAGAG), and GATA (WGATAR), were analyzed in the *KMO* alternative promoters (Yang et al., 2007; Al-Obaide et al., 2015, 2016; Marbach-Bar et al., 2016). JASPAR 2018 database was used to search for the TFBSs of ZNF143 and NRF1 (Khan et al., 2018). CGIs in the alternative promoters identified following the reported parameter set and equation reported by Gardiner-Garden and Frommer (1987) and CGI tools (Supplementary Table S1). The dinucleotide base-stacking energy values were calculated from values of dinucleotide base-stacking energy provided by Ornstein et al. (1978).

### Identification of lncRNA-miR and *KMO*-MRE

The unreported microRNA (miR) sequences of the predicted uncharacterized lncRNA LOC105373233 ncRNA, NCBI Reference Sequence: XR_949327.1, were identified by the miRBase search tool (Griffiths-Jones et al., 2006; Kozomara and Griffiths-Jones, 2014). The miR recognition elements (MREs) in the *KMO* mRNA, NM_003679.4 identified by RNA22 v2 miR target detection tool (Miranda et al., 2006). The miR binds to the target MRE.

### Statistical Analysis

The statistical analyses of the obtained results were achieved using Excel software. The *t*-test was used for analysis of two independent samples, whereas one-way ANOVA was used to analyze the differences among group means of more than two examples. A significant difference was shown by *p* ≤ 0.05.

## Results

### Kynurenic Acid Metabolic Pathway and Genomic Context of *KMO* and ncRNA Uncharacterized LOC105373233 Loci

**FIGURE 1:**
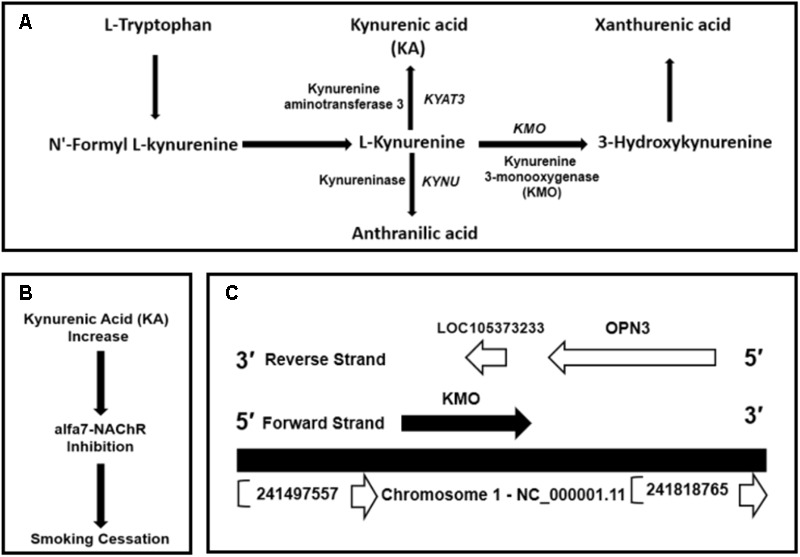
**(A)** Kynurenic acid (KA) is the product of kynurenine pathway, the central metabolite of the tryptophan metabolism. **(B)** Inhibition of kynurenine 3-monooxygenase by Ro 61-8048 or by modulating *KMO* expression could increase the levels of KA. An increase in KA can inhibit α7nAChRs. **(C)** Genomic context of *KMO* gene located on the forward strand at GRCh38/hg38 genomic coordinates: chr1: 241,531,883-241,595,647 and convergently paired to ncRNA uncharacterized LOC105373233 locus mapped on reverse strand at genomic coordinates: chr1:241,585,025-241,588,836. The LOC105373233 locus located at *KMO* 3**′**-region complement strand.

Kynurenic acid is the product of kynurenine pathway (**Figure 1A**). Kynurenine 3-monooxygenase encoded by *KMO* gene is a crucial enzyme in the metabolic regulation of the KA levels. Albuquerque and Schwarcz (2013) and Secci et al. (2017) showed that KA is an antagonist of α7nAChRs in the brain. The Ro 61-8048, a kynurenine 3-monooxygenase inhibitor increases brain KA concentrations and might have applications in the treatment of nicotine addiction (**Figure 1B**) (Röver et al., 1997; Justinova et al., 2013; Secci et al., 2017). Thus, we hypothesized inhibiting or more precisely modulating *KMO* gene expression by genomic and epigenetic approaches could be utilized to enhance the availability of KA.

The human *KMO* locus, NCBI Gene ID: 8564, occupies a region of 63,765 bps on the forward strand of the long arm of chromosome 1 mapped to cytogenetic location 1q43 at GRCh38/hg38 genomic coordinates: chr1: 241,531,883-241,595,647 (**Figure 1C**). The *KMO* mRNA transcript, NM_003679.4, composed of 15 exons (Supplementary Table S2). The *KMO* locus is convergently paired at the reverse strand of 3**′**-region with an ncRNA uncharacterized LOC105373233 locus (**Figure 1C**). The ncRNA locus located at genomic coordinates: chr1:241,585,025-241,588,836 and occupies a genomic region of 3,811 bps, which mapped in the *KMO* region between exons 10 and 13 (Supplementary Figure S1). The uncharacterized LOC105373233 transcript XR_949327 composed of 422 bps. The Ensembl database showed three exons in the genomic space of the LOC105373233 (Supplementary Table S3).

### Differential Activities of Alternative Promoters in Modulating *KMO* mRNA Expressions

We extrapolated *KMO* expression data from the biogps.org to show the variation in *KMO* gene expression at mRNA levels in normal tissues. The analysis of *KMO* mRNA expression in 11 types of human tissues showed variable expressions (Supplementary Figure S2). The statistical analysis indicated that there is a significant difference in the *KMO* expression among the 11 selected adult normal human tissues, *p*-value < 0.05. The observed variation in the *KMO* mRNA expressions in various tissues is an indication of the differential usage of a specific alternative promoter. Thus, alternative promoters can modulate *KMO* expression differentially in various types of cells and tissues according to cellular environments or relevant regulatory factors. This assumption does not ignore the tasks played by other regulatory factors associated with *KMO* expression. We identified 11 *KMO* alternative promoters reported in EPD, TRED, and FANTOM5 databases. EPD and TRED databases showed one and two promoters, respectively, whereas FANTOM 5 database showed eight promoters (Supplementary Table S4). The identified alternative promoters are located on chromosome 1 at the 5′-side of *KMO*-exon 1. Our analysis showed that the 11 alternative promoters’ sequences, AP1–AP11, are overlapping. The *KMO* alternative promoters reported in the FANTOM 5 displayed variable expression in the brain. The p1@*KMO*1 exhibited the highest expression compared with other *KMO* alternative promoters. FANTOM 5 uses CAGE (Cap Analysis Gene Expression) protocol, which identify the exact transcription start sites (TSSs). If the *KMO* is actively expressed because p1@**KMO*1* expression, suitable genomic methods can silence the p1@*KMO*1 (see section “Discussion”). Thus, the promoter can be considered a candidate to lower *KMO* gene expression.

### CpG Island and Transcription Factor Binding Sites in *KMO* Alternative Promoters

**FIGURE 2:**
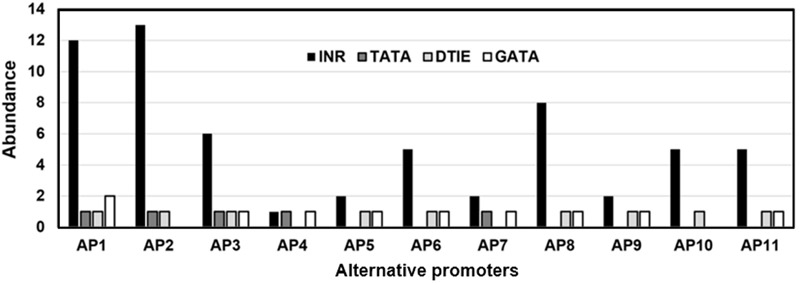
The prevalence of the transcription factor binding sites (TFBSs), TATA-box, INR, and DTIE in the *KMO* alternative promoters. The promoters reported in the TRED (AP1, AP2), EPD (AP3), and PrESSto/FANTOM5 (AP4-AP11) databases.

The *KMO* alternative promoters were investigated for the presence of CGI. None of the analyzed sequences at 5**′**-side of *KMO*-exon-1 showed CGI. The absence of CGI in the *KMO* exon-1 alternative promoters is an indication that these promoters are hosting TATA-box. The incidence of each of the 18 investigated TFBSs in the 11 *KMO* alternative promoters is shown in Supplementary Table S5. Five alternative promoters host TATA-box, 9 have DTIE motifs, and all promoters were rich in INR and Oct4 motifs. We investigated whether there are any statistically significant differences between the three types of motifs associated with transcription initiation, TATA-box, INR, and DTIE. As shown in **Figure 2**, the incidence of TATA-box, INR, and DTIE motifs varied significantly in the 11 identified alternative promoters, *p* < 0.05.

### Structural Characteristics of *KMO* Promoters

**FIGURE 3:**
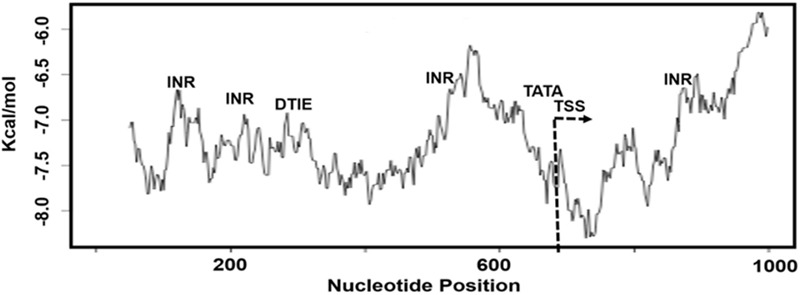
The plot of dinucleotide base pairs stacking energy (kcal/mol) measurement for the sequence *KMO*-TRED 2070 promoter, AP1. TSS, transcription starting site; INR, DTIE, and TATA, TFBS motifs.

The biophysical properties of DNA can disclose the specific locations of TFBS and the CGI along regulatory sequences associated with the process of transcription. We used dinucleotide scales for measurement of base pairs stacking energy values, which we found useful to correlate the structural DNA features with functional sequences, TFBS and CGI, in the alternative promoters. In this context, we studied the dinucleotide base pairs stacking energy along 1000 bps sequence of *KMO* TRED-2070 (AP1) alternative promoter, which overlaps with other promoter sequences (Supplementary Table S4). We observed the promoter characterized by low kcal/mol values, a type of DNA sequence lacks CGI (**Figure 3**). Also, our analysis showed the low kcal/mol values for INR, DTIE, and TATA motifs, which were within range -6.5 and -7.0 kcal/mol. TATA-box motif was mapped nearby TSS region that is easily unstacked.

### The Potential Function of LOC105373233 in the Regulation of *KMO* Expression

**FIGURE 4:**
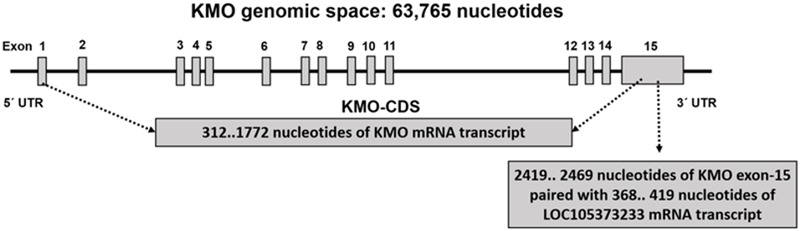
The *KMO* genomic space showing the CDS sequence of *KMO* mRNA transcript NM_003679, and the matching sequence of untranslated *KMO* exon-15 sequence with LOC105373233 ncRNA mRNA sequence.

**Figure 4** and Supplementary Table S2 show the CDS of the *KMO* gene composed of 1461 bases mapped within the *KMO* mRNA transcript, NM_003679.4, composed of 5266 bps. The primary part of exon-15 is untranslated, which suggested the critical function played by the *KMO* 3**′**-UTR region in the regulation of *KMO* expression and imply the presence of regulatory sequences (Supplementary Table S2). The MRE in the *KMO* mRNA is necessary for the miRNA:mRNA prediction, especially in the 3′ UTR. Knowing that the ncRNA loci are sources for miR, we investigated the possible association of the uncharacterized LOC105373233 locus, which encodes an ncRNA, in the regulation of *KMO* expression. We explored the transcripts sequence complementarities of the two convergent loci. NCBI-Nucleotide database showed one uncharacterized LOC105373233 ncRNA transcript, XR_949327.1 predicted by automated computational analysis compared to five transcripts for *KMO* locus; four predicted and one is known mRNA transcript. The predicted ncRNA transcript, namely, LOC105373233 variant transcript XR_949327.1 of 422 bps used in the alignment analysis with *KMO* mRNA transcript NM_003679.4. We identified a sequence in the transcript XR_949327.1 composed of 52 bps showed 75% similarity with the matching *KMO* NM_003679.4 sequence (Supplementary Figure S3). The pairwise sequence alignment analysis suggested the potential formation of a heteroduplex between the 3**′**-UTR region of *KMO*-exon 15 and miR sequence from the ncRNA LOC105373233 transcript, XR_949327.1. The data in **Figure 4** and Supplementary Table S2 also show the matching regions between LOC105373233 ncRNA and the *KMO* 3**′** UTR region. Furthermore, we used miRbase tool to detect the presence of the miR in the ncRNA uncharacterized LOC105373233 transcript, NCBI Reference Sequence: XR_949327.1. The two identified miR sequences, referred to by us hsa-LOC105373233-1 and hsa-LOC105373233-2, are comparable to the human (Homo sapiens) hsa-miR-5096 and hsa-miR-1285-3p respectively (**Table 1**). The identified miR sequences bind target MRE of *KMO* mRNA NM_003679.4; the analysis carried out by the RNA22 v2 tool. The obtained low *p*-values, 3.89E-1-1.43E-3, an indication of a higher chance that the *KMO* mRNA NM_003679.4 contains a valid MRE.

**Table 1:** The miRBase used to search for miR sequences hosted in uncharacterized LOC105373233 ncRNA, NCBI Reference Sequence: XR_949327.1.

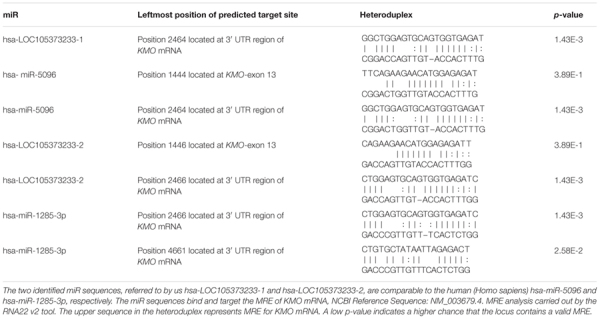

### Identification of Intragenic Promoter With Potential Bidirectional Activity

We hypothesized the presence of an intragenic promoter in the overlapping genomic space of *KMO* in the forward strand that complements ncRNA uncharacterized LOC105373233 in reverse strand. We used the Neural Network Promoter Prediction (Reese, 2001) and Promoter2.0 Prediction Server (Knudsen, 1999) and identified a predicted promoter, which is expected to be involved in the expression uncharacterized ncRNA LOC105373233 locus. The predicted promoter includes a highly likely prediction of TSS with a score of 1.219 shown in Supplementary Figure S1. We investigated the unique regulatory features of the identified promoter sequence occupies 1680 bps in the genomic surroundings up and downstream of the TSS (Supplementary Figure S1). The promoter’s sequence is located at chr1:241,585,594-241,587,273 and includes INR, DTIE, HIF, GATA, and P53 motifs and lacks TATA-box (Supplementary Table S5). A CGI identified in the identified promoter sequence (Supplementary Figure S1). The CGI is of 221 nucleotides located between *KMO*-exon 11 and *KMO*-exon 12 mapped at chr1: 241,587,041-241,587,261. The sequence characterized by the presence of consensus motifs for DNA binding sites of a small group of transcription factors found in bidirectional promoters that included NRF1 and ZNF143, suggesting a possible bidirectional activity (Supplementary Table S5). The predicted intragenic promoter with potential bidirectional activities referred to by us *KMO*-BD. It remained to provide experimental evidence for the transcriptional function of the predicted promoter in the expression of ncRNA LOC105373233 and *KMO* loci (see section “Discussion”).

## Discussion

Understanding the molecular basis of nicotine addiction is a prerequisite to evaluate the effectiveness of protective measures. Analysis of genes and their regulatory factors associated with nicotine addiction provides novel molecular approaches to applying preventive procedures to reduce tobacco smoking harm by supporting smokers to quit and thwart non-smokers from smoking. Several genes found unequivocally associated with nicotine addiction and treatment, namely, *CHRNA3, CHRNB4, CHRNA5, NRXN1*, and *CYP2A6* (Nussbaum et al., 2008; Wassenaar et al., 2011; Broms et al., 2012; Chen et al., 2014; Zhu et al., 2014; Pérez-Rubio et al., 2016). Identifying new genes can give a new supportive approach to the problem. Our investigation of *KMO* locus showed potential use of novel molecular methods in the treatment of tobacco initiation and cessation. The *KMO* gene is pivotal in regulating the availability of KA (Albuquerque and Schwarcz, 2013; Secci et al., 2017). The KA is a neuroprotective and immunomodulating metabolite (Chen and Guillemin, 2009; Bagasrawala et al., 2016; Wirthgen et al., 2018) and inhibits the α7nAChRs in the CNS (Albuquerque and Schwarcz, 2013; Secci et al., 2017). Also, changes in *KMO* expression or activity may contribute to the development of neurodegenerative, neuropsychiatric, and neurodevelopmental diseases (Tan et al., 2012; Parrott and O’Connor, 2015; Smith et al., 2016). Since the KA exhibits a poor penetration of the blood-brain barrier (Fukui et al., 1991; Varga et al., 2016), the modulation of *KMO* expression by RNA interference (RNAi) remains of interest to solve the problem of KA availability in the brain (Mathupala, 2009; Kruspe and Giangrande, 2017). Thus, investigating the influence of genomic and epigenomic regulatory factors in *KMO* expression provides further support to introduce novel personalized medicine approaches for the treatment of individuals vulnerable to nicotine initiation and addiction (Chen and Bierut, 2013; Chen L.S. et al., 2018).

The regulation of *KMO* transcription process and involvement of regulatory elements associated with the process is still poorly understood. We observed in the genomic databases 11 alternative promoters for this gene with variable expression activities. Variable expression of a gene in tissues linked to specific features of the regulatory sequences of the alternative promoters (Liu and Fischer, 1996; Al-Obaide et al., 2015). Our bioinformatic analyses supported this assumption and showed the degree of difference of expression of *KMO* in various human tissues including the human brain linked to the type of *KMO* alternative promoter engaged in the transcription process. In this study, we showed the *KMO* alternative promoters characterized by the absence of CGI. Thus, *KMO* gene is not prone to silencing by CGI methylation (Deaton and Bird, 2011). A recent study showed that genes with and without CGI organized as separated clusters within the genome (Beck et al., 2018). Our thorough analysis of 18 TFBSs revealed three common motifs in the alternative promoters, TATA-box, INR, and DTIE. The incidence of the motifs varied significantly in the 11 identified alternative promoters, *p* < 0.05. Furthermore, the structural characteristics of regulatory sequences can affect the transcription process (Kumar and Bucher, 2016). It is possible to show the difference in the structure of the regulatory sequences by measurements of the dinucleotide stacking energy values. The *KMO* promoters’ sequences showed a co-localized specific pattern of dinucleotide stacking energy associated with transcription starting site (TSS) and the TFBS motifs. The dinucleotide stacking energy can provide approximations regarding the distribution of TFBSs along the sequences of the *KMO* alternative promoters. Also, the measurements of the stacking energy can show the degree of stability and dissociation patterns of the double helix along the regulatory sequence. Previous studies showed the correlation between the specific regulatory sequence and the range of dinucleotide base-stacking energy (Ornstein et al., 1978; Al-Obaide et al., 2015), such correlation observed in the current study. The thermodynamic stability of double-stranded sequences is of importance for understanding the molecular details of gene expression (Häse and Zacharias, 2016). We speculate that the structural features might be the cause of the selective use of one of the alternative promoters by the gene regulatory machinery that triggers off high expression of *KMO* mRNA. We noticed that the p1@*KMO*1 alternative promoter is more active in the brain compared with other *KMO* promoters. It is possible to suggest that p1@*KMO*1 is a candidate to lower *KMO* expression by suitable silencing methods, for example, RNAi, antisense oligonucleotides (ASOs), and CRISPR/Cas9 knockdown (Mathupala, 2009; Kruspe and Giangrande, 2017; Ghosh and Tabrizi, 2017). However, this conclusion does not exclude the functions of additional regulatory factors associated with *KMO* expression besides alternative promoters, for example, miR and ncRNAs.

Another significant finding is identification of an intronic promoter with potential bidirectional activity located at the 3′-region of *KMO* genomic space, which might take part in the control of the expression of ncRNA LOC105373233 and *KMO* loci. The discovered promoter showed the potential to guide the transcription of two loci in a convergent configuration. This type of transcription represented 25% of gene pairs in fungi (Kensche et al., 2008) and reported in mammals (Tran et al., 2003). However, transcription in a divergent manner is the dominant type (Katayama et al., 2005; Engström et al., 2006; Li et al., 2006; Yang and Elnitski, 2008; Seila et al., 2009). Most bidirectional promoters are GC rich and exhibit CGI (Trinklein et al., 2004; Orekhova and Rubtsov, 2013). Additionally, the bidirectional promoters contain consensus sequences of DNA binding sites (motifs) of a small group of transcription factors including NRF1 and ZNF143, while many known transcription factor consensus binding motifs are underrepresented in bidirectional promoters (Lin et al., 2007; Anno et al., 2011). Our analysis showed the predicted bidirectional promoter hosted CGI and the bidirectional specific transcription factors motifs, NRF1 and ZNF143. Our data suggest the possibility of involvement of the identified 3**′**-end in the transcription process of *KMO* from the 3**′**-end. Although transcription starts from the 5**′** end, there are crosstalk between mRNA 3**′**-end processing and transcription initiation (Mapendano et al., 2010). Also, the 3**′**-end regions have a unique and sequence dependent effect on gene expression (Shalem et al., 2013).

The most significant finding reported in this study is the potential function of the predicted uncharacterized ncRNA LOC105373233 locus in the regulation of *KMO* expression. We showed the likelihood of formation of RNA-RNA heteroduplexes between the transcripts of the convergent loci, LOC105373233 ncRNA, and the *KMO* mRNA 3**′** UTR region. Ensembl database shows the biotype of LOC105373233 is long non-coding RNA (lncRNA). The lncRNAs are source for various types of miRNAs (Saini et al., 2007; Dykes and Emanueli, 2017; Chen X. et al., 2018). In this study, we showed that the LOC105373233 ncRNA NCBI Reference Sequence: XR_949327.1 is the source of two discovered miR bind target *KMO*-MRE at 3**′** UTR region. The *KMO*-MRE position is at the identified RNA-RNA heteroduplexes region composed of 52 bps. The two types of miRs are comparable to the human (Homo sapiens) hsa-miR-5096 and hsa-miR-1285-3p. Our data suggested potential downregulation of *KMO* expression by the miR hosted in the convergently paired uncharacterized ncRNA LOC105373233 locus. However, if the identified miRNAs deregulated, the *KMO* escapes miRNA-mediated repression. The mechanism by which miRNA control gene expression is a novel sophisticated process. Briefly, miRNAs are processed from their hairpins and loaded into the Argonaute (AGO) proteins in effector complexes known as miRNA-induced silencing complexes (miRISCs). Consequently, the miRNA serves as a guide for this complex to the target mRNA where it induces translational repression (Bartel, 2009; Hausser and Zavolan, 2014; Issler and Chen, 2015). The miRISCs promote endonucleolytic cleavage of fully complementary targets or translational repression, mRNA deadenylating, and exonucleolytic decay of targets with partial complementarity. It is believed the primary function of most of the ncRNAs in mammals downregulating the level of corresponding mRNA expression (Song et al., 2012; Eades et al., 2014; Uesaka et al., 2014). Also, ncRNAs are tissue specific (Jiang et al., 2016).

An exciting prospect of our study identification of several regulatory modes involved in modulation of *KMO* gene expression. For instance, the study suggested that the *KMO* variable expression is the result of alternative promoters. The novel finding of this study is the potential role of the ncRNA uncharacterized LOC105373233 locus in the regulation of *KMO* expression. The *KMO* downregulation by lncRNA LOC105373233 might increase KA levels that inhibit α7nAChRs. The data are likely to advance our knowledge about the function and application of this gene in tobacco smoking cessation and can have applications for individuals more vulnerable to nicotine addiction.

## Author Contributions

HAA, A-SA-S, and MA-O contributed equally to the design and writing of the paper. HWA and SA-H helped in the analysis.

## Conflict of Interest Statement

HA, A-SA-S, and MA-O have no competing interests with Biomedica, LLC. The other authors declare that the research was conducted in the absence of any commercial or financial relationships that could be construed as a potential conflict of interest.
