# Supplementary material for: Kynurenine 3-Monooxygenase Gene Associated With Nicotine Initiation and Addiction: Analysis of Novel Regulatory Features at 5′ and 3′-Regions
Source: Front Genet. 2018 Jun 13;9:198. doi: 10.3389/fgene.2018.00198 (PMC6008986; doi:10.3389/fgene.2018.00198)
Supplement: Supplementary file 1 [file Table_1.docx]

**Table 1.** The miRBase used to search for miR sequences in uncharacterized LOC105373233 ncRNA, NCBI Reference Sequence: XR_949327.1. The two identified miR sequences, referred to by us hsa-LOC105373233-1 and hsa-LOC105373233-2 are comparable to the human (Homo sapiens) hsa-miR-5096 and hsa-miR-1285-3p respectively. The miR sequences bind and target the MRE of *KMO* mRNA, NCBI Reference Sequence: NM_003679.4. MRE analysis carried out by the RNA22 v2 tool. The upper sequence in the heteroduplex represents MRE for *KMO* mRNA. A low p-value indicates a higher chance the locus contains a valid MRE.

| miR | Leftmost position of predicted target site | Heteroduplex | p value |
| --- | --- | --- | --- |
| hsa-LOC105373233-1 | Position 2464 located at 3' UTR region of KMO mRNA | GGCTGGAGTGCAGTGGTGAGAT \| \|\|\|\| :\|\| \|\|\|\|\|\|:\|: CGGACCAGTTGT-ACCACTTTG | 1.43E-3 |
| hsa- miR-5096 | Position 1444 located at KMO-exon 13 | TTCAGAAGAACATGGAGAGAT  \|\| \|\|\|\|\|\|\| \|\|:\|: CGGACTGGTTGTACCACTTTG | 3.89E-1 |
| hsa-miR-5096 | Position 2464 located at 3' UTR region of KMO mRNA | GGCTGGAGTGCAGTGGTGAGAT \| \|\|\|: :\|\| \|\|\|\|\|\|:\|: CGGACTGGTTGT-ACCACTTTG | 1.43E-3 |
| hsa-LOC105373233-2 | Position 1446 located at KMO-exon 13 | CAGAAGAACATGGAGAGATT  \|\|\|\|\|\|\| \|\|:\|:: GACCAGTTGTACCACTTTGG | 3.89E-1 |
| hsa-LOC105373233-2 | Position 2466 located at 3' UTR region of KMO mRNA | CTGGAGTGCAGTGGTGAGATC \|\|\|\| :\|\| \|\|\|\|\|\|:\|:\| GACCAGTTGT-ACCACTTTGG | 1.43E-3 |
| hsa-miR-1285-3p | Position 2466 located at 3' UTR region of KMO mRNA | CTGGAGTGCAGTGGTGAGATC \|\|\|\| :\|\|: :\|\|\|\|\|\|:\| GACCCGTTGTT-TCACTCTGG | 1.43E-3 |
| hsa-miR-1285-3p | Position 4661 located at 3' UTR region of KMO mRNA | CTGTGCTATAATTAGAGACT \|\|\| \|\| \|:\|\| \|\|\|\|\|: GACCCGTTGTTTCACTCTGG | 2.58E-2 |
