# Supplementary material for: Kynurenine 3-Monooxygenase Gene Associated With Nicotine Initiation and Addiction: Analysis of Novel Regulatory Features at 5′ and 3′-Regions
Source: Front Genet. 2018 Jun 13;9:198. doi: 10.3389/fgene.2018.00198 (PMC6008986; doi:10.3389/fgene.2018.00198)
Supplement: Supplementary file 2 [file Table_2.DOCX]

Supplementary Material

**Kynurenine 3-Monooxygenase Gene Associated with Nicotine Initiation and Addiction: Analysis of Novel Regulatory Features at 5' and 3'- Regions**

**Hassan A. Aziz^1^, Abdel-Salam Gomaa Abdel-Salam^1*^, Mohammed A. Ibrahim Al-Obaide^2^, Hytham W. Alobydi^3^, Saif Al-Humaish^3^**

*** Correspondence:** Corresponding Author: abdo@qu.edu.qa

**Table S2.** The 15 exons of the human *KMO* NM_003679.4 mRNA composed of 5266 bps. The *KMO*-CDS sequence highlighted red. The box shows the *KMO* 3' UTR region complementary to the ncRNA LOC105373233. The *KMO*-MRE underlined in the box. The microRNA recognition elements (MRE) identified by the RNA22 v2 tool (Miranda et al., 2006).

| KMO mRNA | Sequences of exons | Span in mRNA | Map location |
| --- | --- | --- | --- |
| Exon 1 | GCTTAAAAATTTCTGTGTCTTACACAGAAGATAGAAAAAATAGAGTGTCTCCAATTGGATGGATTTTTTA AAAAATTTGGTTATTGTAATGGATTTATTTTTTCTTAGAGCTGAGCTGATTGTACTTTGGCCAACTAATG GGTTAATACTGTCAAGGGAAATTAGCCCTGACTAAACATTGCCGCTGGCTCATGAATGCACTAGGCTTGG GGCAGTATAAAAACTCAGAGAAATCAGTGTGTAGGAGACACAGAAATCAGTGTCACTCAGTGACAGAAGC AACAATAATTGTGAAAAATACTTCAGCAGTTATGGACTCATCTGTCATTCAAAGGAAAAAAGTAGCTGTC  ATTGGTGGTGGCTTG | 1-365 | Chr1:241532134-241532498 |
| Exon 2 | TTGGCTCATTACAAGCATGCTTTCTTGCAAAGAGGAATTTCCAGATTGATGTATATGAAGCTAGGGAAG | 366-435 | Chr1:241548829-241548898 |
| Exon 3 | ATACTCGAGTGGCTACCTTCACACGTGGAAGAAGCATTAACTTAGCCCTTTCTCATAGAGGACGACAAGC  CTTGAAAGCTGTTGGCCTGGAAGATCAG | 436-533 | Chr1:241549677-241549774 |
| Exon 4 | ATTGTATCCCAAGGTATTCCCATGAGAGCAAGAATGATCCACTCTCTTTCAGGAAAAAAGTCTGCAATTC  CCTATGGGACAAAGTCTCAG | 534-623 | Chr1:241550955-241551044 |
| Exon 5 | TATATTCTTTCTGTAAGCAGAGAAAATCTAAACAAGGATCTATTGACTG | 624-672 | Chr1:241555612-241555660 |
| Exon 6 | CTGCTGAGAAATACCCCAATGTGAAAATGCACTTTAACCACAGGCTGTTGAAATGTAATCCAGAGGAAGG  AATGATCACAGTGCTTGG | 673-760 | Chr1:241560665-241560752 |
| Exon 7 | ATCTGACAAAGTTCCCAAAGATGTCACTTGTGACCTCATTGTAGGATGTGATGGAGCCTATTCAACTGTC  AGATCTCACCTGATGAAGAAACCTCGCTTTGATTACAGTCAGCAGTACATTCCTCATGGGTACATGGAGT  TGACTATTCCACCTAAGAACGGAGAT | 761-926 | Chr1:241562167-241562332 |
| Exon 8 | TATGCCATGGAACCTAATTATCTGCATATTTGGCCTAGAAATACCTTTATGATGATTGCACTTCCTAACA  TG | 927-998 | Chr1:241564987-241565058 |
| Exon 9 | AACAAATCATTCACATGTACTTTGTTCATGCCCTTTGAAGAGTTTGAAAAACTTCTAACCAGTAATGATG  TGGTAGATTTCTTCCAGAAATACTTTCCGGATGCCATCCCTCTAATTGGAGA | 999-1120 | Chr1:241566491-241566612 |
| Exon 10 | GAAACTCCTAGTGCAAGATTTCTTCCTGTTGCCTGCCCAGCCCATGATATCTGTAAAGTGCTCTTCATTT  CACTTTAAATCTCACTGTGTACTGCTGGGAGATGCAGCTCATGCTATAGTGCCGTTTTTTGGGCAAGGAA  TGAATGCG | 1121-1268 | Chr1:241568500-241568647 |
| **Exon 11** | ***GGCTTTGAAGACTGCTTGGTATTTGATGAGTTAATGGATAAATTCAGTAACGACCTTA*** | 1269-1326 | Chr1:241586679-241586736 |
| Exon 12 | GTTTGTGTCTTCCTGTGTTCTCAAGATTGAGAATCCCAGATGATCACGCGATTTCAGACCTATCCATGTA  CAATTACATAGAG | 1327-1409 | Chr1:241588748-241588830 |
| Exon 13 | ATGCGAGCACATGTCAACTCAAGCTGGTTCATTTTTCAGAAGAACATGGAGAGATTTCTTCATGCGATTA  TGCCATCGACCTTTATCCCTCTCTATACAATG | 1410-1511 | Chr1: 241590012-241590113 |
| Exon 14 | GTCACTTTTTCCAGAATAAGATACCATGAGGCTGTGCAGCGTTGGCATTGGCAAAAAAAG | 1512-1571 | Chr1:241590204-241590263 |
| Exon 15 | GTGATAAACAAAGGACTCTTTTTCTTGGGATCACTGATAGCCATCAGCAGTACCTACCTACTTATACACT  ACATGTCACCACGATCTTTCCTCCGCTTGAGAAGACCATGGAACTGGATAGCTCACTTCCGGAATACAAC  ATGTTTCCCCGCAAAGGCCGTGGACTCCCTAGAACAAATTTCCAATCTCATTAGCAGGTGATAGAAAGGT  TTTGTGGTAGCAAATGCATGATTTCTCTGTGACCAAAATTAAGCATGAAAAAAATGTTTCCATTGCCATA  TTTGATTCACTAGTGGAAGATAGTGTTCTGCTTATAATTAAACTGAATGTAGAGTATCTCTGTATGTTAA  TTGCAATTACTGGTTGGGGGGTGCATTTTAAAAGATGAAACATGCAGCTTCCCTACATTACACACACTCA  GGTTGAGTCATTCTAACTATAAAAGTGCAATGACTAAGATCCTTCACTTCTCTGAAAGTAAGGCCCTAGA  TGCCTCAGGGAAGACAGTAATCATGCCTTTTCTTTAAAAGACACAATAGGACTCGCAACAGCATTGACTC  AACACCTAGGACTAAAAATCACAACTTAACTAGCATGTTAACTGCACTTTTCATTACGTGAATGGAACTT  ACCTAACCACAGGGCTCAGACTTACTAGATAAAACCAGAAATGGAAATAAGGAATTCAGGGGAGTTCCAG  AGACTTACAAAATGAACTCATTTTATTTTCCCACCTTCAAATATAAGTATTATCATCTATCTGTTTATCG  TCTATCTATCTATCATCTATCTATCTATCTATCATCTATCTATCTATCTATCTATCTATCTATCTATCTA  TCTATCT**CTATTTATTTATGTATTTAGAGATCAGGTCTCACTCTGTTGACCAGGCTGGAGTGCAGTGGTG**  **AGAT**CTGGGTTCACTGCAACCTCTGCCTCCTGGGCTCAAGCAATCCTCCCACTTCAGCCTCCCAAATAGC  TGGGGCTACCATGGTATTTTTCAGTAGAGACCGGGTCTTGCCATGCTGCCCAGGCCAGTCTCAAACTCCT  GGCCTCATGTGATCTGCCCACCTCAGCCTCCCAAAGTACAGGGATTAGAGTTGTGAGCCACCGCTGCCAG  CCCAGAGTTACCCTCTAAAGATAAGAAAAAGGCTATTAATATCATACTAAGTGAAGGACAGGAAAGGGTT  TTATTCATAAATTAAATGTCTACATGTGCCAGAATGGAAAGGAAACAAGGGGAGACAACTTTTATAGAAA  TACAAAGCCATTACTTTATTCAATTTCAGACCCTCAGAAGCAATTTACTAATTTATTCTTCGACTACATA  CTGCAGCAGAACCAGCAATACACTTGATTTTTAAAAGCACATTTAGTGAAATGTTTTCTTTGGTTCATCC  TTCTTTAACAGGCTGCTGAGTCACTCAGAAATCCTTCAAACATGATTAATTATGAAGATGAAACACTAGA  GTCATATAAGAAATAAAAATTGGGCAATAAAATAAAATGATTCAGTGTTTCTTTTCTATATTGTCAATGA  AAACCTTGAGTTCTAATAATCCATGTTCAGTTTGTAGGGAAAGAAAAAATAATTTTTCCTTCTACCCACT  TTAGGTTCCTTGGCTGGGGCCCCTATAACAAAAGACAGATTGACAAGAGAAAAACAAACATAAATTTATT  AGCGGGTATATGTAATATATATGTGGGAAATACAGGGGAATGAGCAAATCTCAAAGAGCTGGCGTCTTAG  AACTCCCTGGCTTATATAGCATCGACAAAGAACAGTAAATTTTTAGAGAAACAACAAAACAAAGAAAAAG  AGCTTTGAGTCTGTAGGGGCAGCAATTTGGGGGAAGCAAATATATGGGAGTTTGCCTTGTAGATTCCTCT  GGTGCTGGTCTCCAGGCTGACAAGGATTCAAAGTTGTCTCTGAAACTCCTCTTTGTCATACTGCACATAT  AAAACGTCTTTTGTTTCCAACAAGAGGATTTTCTTTTTCATTCTAGAATTATCTCCTTGATAACTTGATC  AGATATAGGACATGACACTGAATAGAGTCCAACAGTACAAAAAAAATTCAGTATGTTCTAGCTACTTCAC  ACATGTGTACGCGACAGTTATTTTTACAGTAAGGTATTTTCGAGAAAAATGCATTACGTGTTTTGGAAAA  TAGAGTAATTTAAAAAATATATTTGAAATGAAAATCTCCAACACATTAGAAGATGATGATGTTAGATGCC  CATCGTGTGCCACAAGTGGTTTTTTCATTATGTAAAGCACCCGTTGAATTAAAAGAATTTGTTTTTGTTC  AACCTCTTCCTGAGGCCCAAGAGCATATGGGCAATTCGGATTTCCTGCTGGACCACAAGGTTCTGTTGAT  ATTACATAGAACGGGTATTCCAGACACTTCTTATGATGAAAGTCCAAAAGTGGCATCCAATTTAAGGCCC  CATCTTTCGTTGCCATTCTTCATTCCTACAAAGGACGAACTTGGATTACATCAACTTTGGACCCATTGGT  TTTGTCGCTGTCGTCAACTGACAGTGATTCATCACTGGTGATGATAAAAATGATGGAAGAAGAGTTGAAA  GTCACTTTTTTCTTTGGCCTGTCCCCATCTTTCTGTGACATCACAATGGGTCTGATCTGCATTTCACTTC  CAGCTGCTGGTAGGTCTTTAGCAGGCCTCTGGCACCTCAGCAGTCGGAGGCACAGAAGCTGCAAAAGGGA  TCTTCGAAACTGGGCAGAGAAAAAATAAAGTGGAATATTAAGTAAAAGTTGGGCACTAATCTGGATTAAC  ATTCGAGGAAATCAGTTGAGCTGAATTTAAGTTGTTTTTTGTTTGTTAGCAGGTGTGGATGTGGGGTTAT  GTGGTCATGCTCAGATCTACCTAAATCACCCCAGAGCTTTATGTCTTTTATTCATTCTAATTCTTATTAA  CCGGAATATGTAGGACCATTTCAATACCTTGTAATCCTCCAAGCTTCAATCTGCACACACTTTCTATGAG  GGCAGGTACAACTATTAAGAGATTTTGAACATTAAGTTAGTCCACAAATATTCAGTGGGCATCTACTAGG  TGACAGCCACTGTGCTATAATTAGAGACTTTTTACTATAAGCATCAAAAACAGATAAGGCTCTTCCTGGC  AGAGTTTACAGCCTGGTGTACTTGCTAATGTCTCTTTAATTAGGTGAAGAATTTTTTTTTTCTATCGAAA  TTACTAATCAGTTGGGGAAAAAAATACTATAGCAGACAGCACTAATGTCATCAACAAACATTGTTCTTCT  CCGTGTCCTGGGTACAACATCGAATAATATTTCTTGGCCTCCTTTCCGCTTCTCCTCTCTGCTGTTCCTC  TCTACAAGAACCTGGGAGGCCAACGCCTAAAGATCATAATATCACACAATGGAAGGAACCTAGATTCCTA  AATGACTGCATAGGACAGATCCCATCTCCTCCACCCAATACATTATTAGACTGAACTGTGACCTGAAATG  AGCAATAAACTCTGTATTAATTCACTGAAATGTTGGGGTTGCTTGTTATAGTAGTCGGTCCATCATGACC  AGTAAAACATAAATCAAAAGTTAATGTAATTGTTATCCCATTATTTAGAGCGAAATAAATGTTGAATATA  TGGACTTTCTCAGATTAGGAAATACCAATTAAAAATATAATAAATAGCTACATTG | 1572-5266  (3695 bps) | Chr1:241591953-241595647 |

**
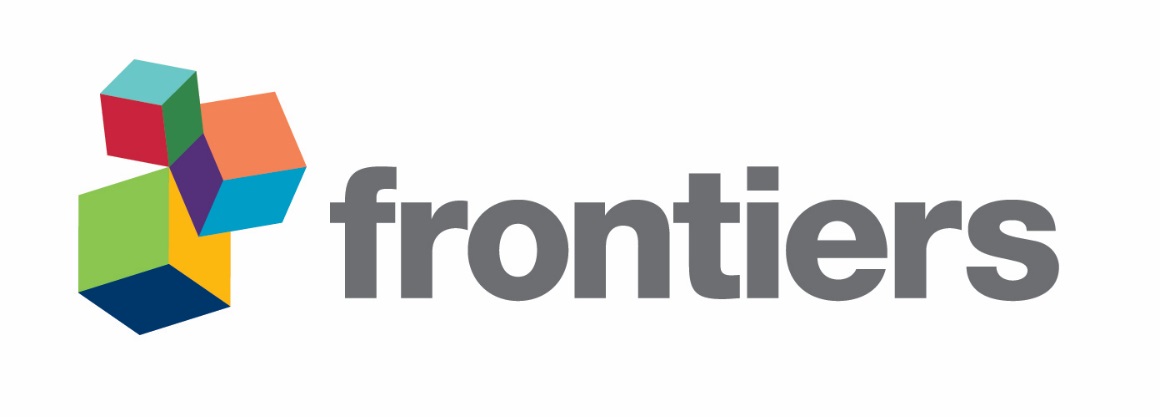
**
