# Supplementary material for: Kynurenine 3-Monooxygenase Gene Associated With Nicotine Initiation and Addiction: Analysis of Novel Regulatory Features at 5′ and 3′-Regions
Source: Front Genet. 2018 Jun 13;9:198. doi: 10.3389/fgene.2018.00198 (PMC6008986; doi:10.3389/fgene.2018.00198)
Supplement: Supplementary file 3 [file Table_3.DOCX]

Supplementary Material

**Kynurenine 3-Monooxygenase Gene Associated with Nicotine Initiation and Addiction: Analysis of Novel Regulatory Features at 5' and 3'- Regions**

**Hassan A. Aziz^1^, Abdel-Salam Gomaa Abdel-Salam^1*^, Mohammed A. Ibrahim Al-Obaide^2^, Hytham W. Alobydi^3^, Saif Al-Humaish^3^**

*** Correspondence:** Corresponding Author: abdo@qu.edu.qa

**Table S3.** The exons of the predicted ncRNA uncharacterized LOC105373233 locus XR_949327.1. The box shows the uncharacterized LOC105373233 matching sequence to the KMO 3' UTR region. The exons data from the Ensembl-sequence database.

| Exons | Sequences of exons | Span in mRNA | Map location |
| --- | --- | --- | --- |
| Exon 1 | ACTCACCTCTATGTAATTGTACATGGATAGGTCTGAAATCGCGTGATCATCTGGGATTCTCAATCTTGA  GAACACAGGAAGACACAAACCT | 1-91 | Chr1: 241588746-241588836 |
| Exon 2 | TGCCCCAAACCAGCGTTGACAGTGTGTTCTTATGCATAAACCCTCTATCAACCTCCAACGTCATGTGTAA  ACCAGTCACGCGAGCACAAGGAAAGGAGGAAACAAAATATCTCTGGCTCCTGAGCATCTGAGAGAGATGA  CTGGGAAATAACCAAAAAAAAAAAAAAAAAAAAAAAAGAAAAAAAAGATGAGAAAATTTGTCCTGGAATT  CTATGATCTCTTCAGACCCAGGCACCAGTATTCTTGGACTTTCTAAAACACCACAGCAAACATATACTAT  GAACTTACTTTACG | 92-385  (294 bps) | Chr1: 241588213-241588506 |
| Exon 3 | TAGAGACGGGGTTTCACCATGTTGACCAGGCTGGCCT | 386-422 | Chr1: 241585024-241585062 |

**
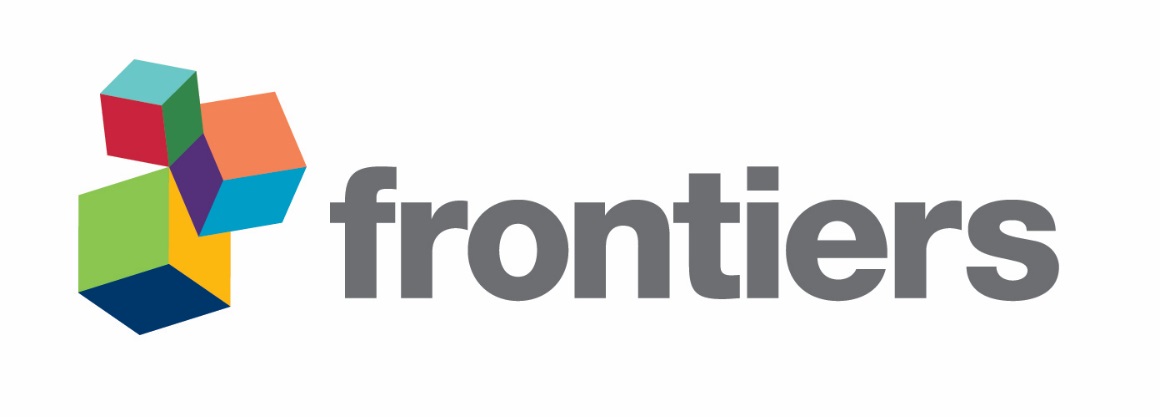
**
