# Supplementary material for: Kynurenine 3-Monooxygenase Gene Associated With Nicotine Initiation and Addiction: Analysis of Novel Regulatory Features at 5′ and 3′-Regions
Source: Front Genet. 2018 Jun 13;9:198. doi: 10.3389/fgene.2018.00198 (PMC6008986; doi:10.3389/fgene.2018.00198)
Supplement: Supplementary file 4 [file Table_4.DOCX]

Supplementary Material

**Kynurenine 3-Monooxygenase Gene Associated with Nicotine Initiation and Addiction: Analysis of Novel Regulatory Features at 5' and 3'- Regions**

**Hassan A. Aziz^1^, Abdel-Salam Gomaa Abdel-Salam^1*^, Mohammed A. Ibrahim Al-Obaide^2^, Hytham W. Alobydi^3^, Saif Al-Humaish^3^**

*** Correspondence:** Corresponding Author: abdo@qu.edu.qa

**Table S4.** The eleven *KMO* alternative promoters reported in the databases. The promoters map locations at the *KMO* 5'-region.

| **Source** | **Alternative promoter ID** | **Symbol** | **Map locations** | **Span (bps)** |
| --- | --- | --- | --- | --- |
| TRED | *KMO*-2070 | AP1 | Chr1: 241531680-241532679 | 1000 |
|  | *KMO*-115569 | AP2 | Chr1: 241531707-241532706 | 1000 |
| EPD | FP002547 *KMO*-1 | AP3 | Chr1: 241531756-241532355 | 600 |
| PrESSto/FANTOM5 | p1@*KMO* | AP4 | Chr1: 241532079-241532479 | 401 |
|  | p2@*KMO* | AP5 | Chr1: 241531834-241532234 | 401 |
|  | p3@*KMO* | AP6 | Chr1: 241531600-241532000 | 401 |
|  | p4@*KMO* | AP7 | Chr1: 241531955-241532355 | 401 |
|  | p6@*KMO* | AP8 | Chr1: 241531584-241531984 | 401 |
|  | p7@*KMO* | AP9 | Chr1: 241531848-241532248 | 401 |
|  | p8@*KMO* | AP10 | Chr1: 241531742-241532142 | 600 |
|  | p9@*KMO* | AP11 | Chr1: 241531641-241532041 | 600 |

**
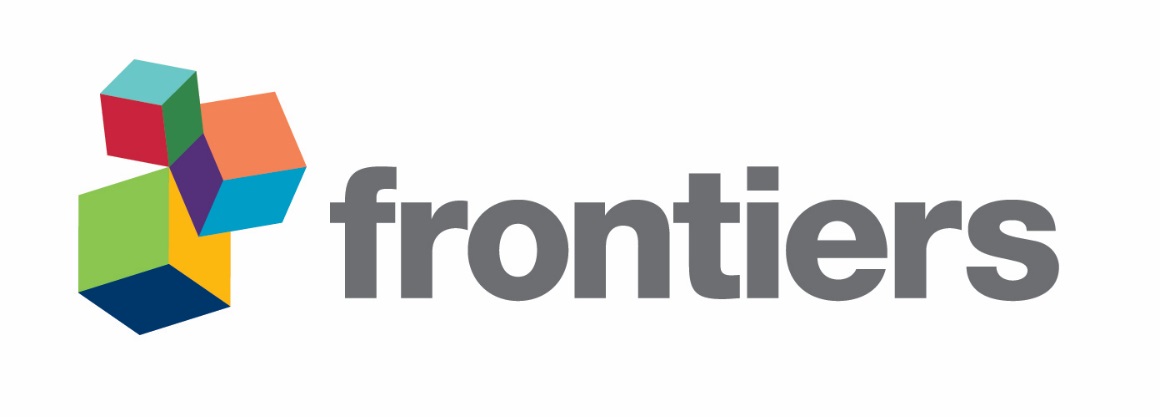
**
