# Supplementary material for: Kynurenine 3-Monooxygenase Gene Associated With Nicotine Initiation and Addiction: Analysis of Novel Regulatory Features at 5′ and 3′-Regions
Source: Front Genet. 2018 Jun 13;9:198. doi: 10.3389/fgene.2018.00198 (PMC6008986; doi:10.3389/fgene.2018.00198)
Supplement: Supplementary file 5 [file Table_5.DOCX]

Supplementary Material

**Kynurenine 3-Monooxygenase Gene Associated with Nicotine Initiation and Addiction: Analysis of Novel Regulatory Features at 5' and 3'- Regions**

**Hassan A. Aziz^1^, Abdel-Salam Gomaa Abdel-Salam^1*^, Mohammed A. Ibrahim Al-Obaide^2^, Hytham W. Alobydi^3^, Saif Al-Humaish^3^**

*** Correspondence:** Corresponding Author: abdo@qu.edu.qa

**Table S5.** The prevalence of the transcription factors motifs in the *KMO* alternative promoters mapped at 5'-side. The promoters are reported in the TRED, EPD and FANTOM5 databases. The identified intragenic *KMO*-BD promoter located at the 3' region of *KMO* genomic space. ZNF143* and NFR1* motifs identified by the JASPAR2018 search tool.

| **TFBSs ID** | **Consensus motifs** | **KMO-BD** | **Alternative promoters** | | | | | | | | | | |
| --- | --- | --- | --- | --- | --- | --- | --- | --- | --- | --- | --- | --- | --- |
|  |  |  | **AP1** | **AP2** | **AP3** | **AP4** | **AP5** | **AP6** | **AP7** | **AP8** | **AP9** | **AP10** | **AP11** |
| INR | YYANWYY | 16 | 12 | 13 | 6 | 1 | 2 | 5 | 2 | 8 | 2 | 5 | 5 |
| TATA | TATAWA | 0 | 1 | 1 | 1 | 1 | 0 | 0 | 1 | 0 | 0 | 0 | 0 |
| DTIE | GBBRDNHGG | 2 | 1 | 1 | 1 | 0 | 1 | 1 | 0 | 1 | 1 | 1 | 1 |
| BRE | SSRCGCC | 0 | 0 | 0 | 0 | 0 | 0 | 0 | 0 | 0 | 0 | 0 | 0 |
| DPE | RGWCGTG | 0 | 0 | 0 | 0 | 0 | 0 | 0 | 0 | 0 | 0 | 0 | 0 |
| M3 | SCGGAAGY | 0 | 0 | 0 | 0 | 0 | 0 | 0 | 0 | 0 | 0 | 0 | 0 |
| M22 | TGCGCANK | 0 | 0 | 0 | 0 | 0 | 0 | 0 | 0 | 0 | 0 | 0 | 0 |
| HIF | RCGTG | 2 | 0 | 0 | 0 | 0 | 0 | 0 | 0 | 0 | 0 | 0 | 0 |
| EBF | CCCNNGGG | 0 | 0 | 0 | 0 | 0 | 0 | 0 | 0 | 0 | 0 | 0 | 0 |
| Sox | WWCAAWG | 0 | 1 | 1 | 0 | 1 | 0 | 0 | 0 | 0 | 0 | 0 | 0 |
| P53 | RRRCWWGY | 1 | 0 | 0 | 0 | 0 | 0 | 0 | 0 | 0 | 0 | 0 | 0 |
| Oct4 | TTTKSWTW | 0 | 5 | 5 | 3 | 2 | 2 | 1 | 2 | 1 | 3 | 1 | 1 |
| PU.1 | GAGGAA | 0 | 0 | 0 | 0 | 0 | 0 | 0 | 0 | 0 | 0 | 0 | 0 |
| Nanog | SRSSATTANS | 0 | 0 | 0 | 0 | 0 | 0 | 0 | 0 | 0 | 0 | 0 | 0 |
| c-myc | CACRTG | 0 | 0 | 0 | 0 | 0 | 0 | 0 | 0 | 0 | 0 | 0 | 0 |
| MAD | CCGNCGCG | 0 | 0 | 0 | 0 | 0 | 0 | 0 | 0 | 0 | 0 | 0 | 0 |
| GAF | GAGAG | 0 | 0 | 0 | 0 | 0 | 0 | 0 | 0 | 0 | 0 | 0 | 0 |
| GATA | WGATAR | 1 | 2 | 0 | 1 | 1 | 1 | 1 | 1 | 1 | 1 | 0 | 1 |
| ZNF143* | - | 1 | 0 | 0 | 0 | 0 | 0 | 0 | 0 | 0 | 0 | 0 | 0 |
| NRF1* | - | 4 | 0 | 0 | 0 | 0 | 0 | 0 | 0 | 0 | 0 | 0 | 0 |

**
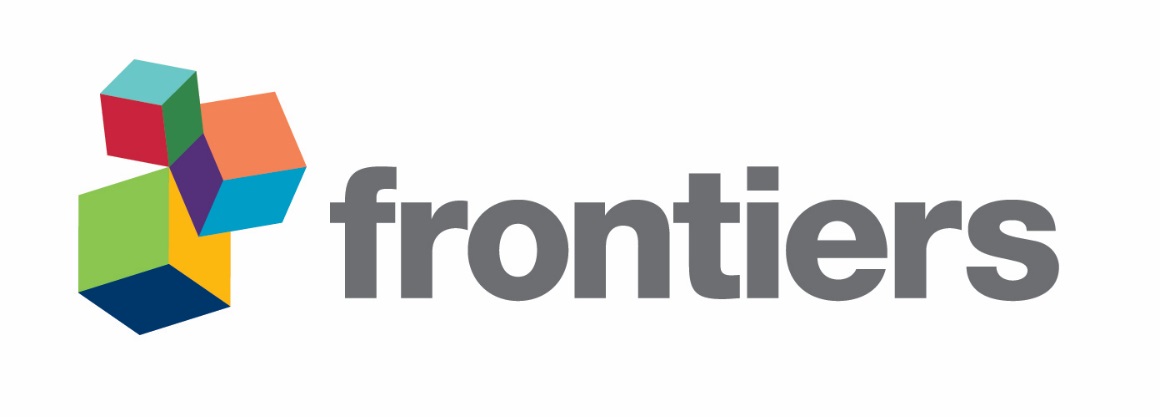
**
