# Supplementary material for: Kynurenine 3-Monooxygenase Gene Associated With Nicotine Initiation and Addiction: Analysis of Novel Regulatory Features at 5′ and 3′-Regions
Source: Front Genet. 2018 Jun 13;9:198. doi: 10.3389/fgene.2018.00198 (PMC6008986; doi:10.3389/fgene.2018.00198)
Supplement: Supplementary file 6 [file Data_Sheet_1.PDF]

Supplementary Material

Kynurenine 3-Monooxygenase Gene Associated with Nicotine Initiation and Addiction: Analysis of Novel Regulatory Features at 5' and 3'-Regions

Hassan A. Aziz[1], Abdel-Salam Gomaa Abdel-Salam[1*], Mohammed A. Ibrahim Al-Obaide[2], Hytham W. Alobydi[3], Saif Al-Humaish[3]

*** Correspondence:** Corresponding Author: Abdo@qu.edu.qa

Figure S1. The *KMO* forward strand showing the complementary DNA sequence of ncRNA uncharacterized LOC105373233 exons and the identified bidirectional promoter (*KMO*-BP), for details see the main text. The uncharacterized LOC105373233 mapped in the *KMO* region between exons 10 and 13.

ACATTAGGTATTTCTCCTAATGCTATCCCTCCCCCATCCCCCAACCCCATGACAGCCCCC

GGTGTGTGATGTTCCCCTTCCTGTGTCCATGTGTTCTCATTGTTCAATTCCCACCTATGA

GTGAGAACATGAGGTGTTTGGTTTTCTGTCCTTGCAACTCATCATCACTGGTCATCAGAG

AAATGCAAATCAAAACCACAATGAGATACCATCTCACACCAGTTAGAAAGGTGATTGTTA

AAAAGTCAGGAAACAACAGGTGCTGGAGAAGATGTGGAGAAATAGGAACACTTTCACACT

GTTGGTGGGAGTGTAAACTAGTTCAACCATT**GTGGAAGACAGTGTGG**CGATTCCTCAAGG

End of ncRNA

ATCTCTTTCTTTTCTACTTTTTCTTTCCAAAGGAAAGCAAAACCTGATTGACAAGGTTGA

GTAAAACTATGAGCTGATTAGATGTTGAACACTACTGCCTTCATGGAAATGTTACTATTT

TGGCCAGAAAAAGAATTCAGAAAAGAAATAGCGTTTAAAAGTTGAACATTCATTTCAAGG

AATCCTAATTTGTGTGTGTGTGTGCACTAATAGCTTCATAATCATTAATCATATATTATT

TTGGTCATTGGCATATATTCCAAGTAAAAATAGCTTTTGAATCTAATCAATTATATTCTC

CATGAAGCAGTGGTAAACATATTGTTTTATAAAAACATAAGACATTAACTGTAGCACAGT

GGATAAGTGCCCAGGACCTGACATTAGAATACTTAGTTTTAAATCCTGGCTCCAGAATTT

ACTGCAAATATATTCTTGTGCAATTTACTTACCTTATCTTTACTTTCTTATCCATAAAAC

TGGAATTTTAGGCCAGGTGTGGTGGCTCACGCCTGTAATCCCAGCACTTTGGGAGACCGA

GACAGGTGGATCACTTGAGGTCAGGAGGTTGAGGCCAGCCTGGTCAACATGGTGAAACCC

CGTCTCTACTGAAAATACAAAAAAAATTAGCTAGGTGTGGTGGTGGGTTCCTGTAGTCCC

ncRNA-exon 3 underlined

AACTACTTGGGAGGCTAAGGCAGGAGAATCGCTTGAACCTGGCAGGTGGAGGTTGCAGTG

AGCCGAGATTGTGCCACTGCACTCCTGCCTGGGTGACAGACCGAGACTGCATTTCAAAAT

TAATTAATTAATAATTAAATTAAATTAAATTGGAATTTTAATAATGTCTACCTCAGAGAG

TTGTGAAGAGTAAATGAATTAACTTAAACAAAAGAATATAAAACTATTATCTCAGCCCCA

TATGGTATCTCTCACTTCTATAGTATTGAGACTATATAATTGATTTGACAAATAAATCTG

CTTGTTTCAGATGTAATCTTTATAATTGATGTGAAAAATATTTTATTTAAAAAATATCTT

AAGGCCGGGCACAGTGGCTCACGCTTGTAATCCCAGCACTTTGGGAGGCCAAGCCAGGCA

GATCACTTGAGGTCAAGAGTTTGAGACCAGTCTGGCCAATGTGGTGAAATGCCATCTCTA

KMO-BP

TAAAAATACAAAAATTAGCTGGGCGTGGTGGTGCACGCCTGTAATCCTGGCTACTTGGGA

GGCTGAGGAAGGAGAATCATTTGAACCTAACAGGTGGAGATTGAAGTGAGCCGAGATTGT

GCTGCACTCCAACCTGAACAACAGAGCAAGACTCCATCTAAAATAAAAAAAAAAGAACAA

AAAAAAAAACCAAAAAGAAAAAAAAATCTTTATACTACCTCTAAAGCTGTAGCTTGAATTC

CTAGGAAACAGGAAACATTGTTTTCAATTCTCCATCCTCTCTGTATTTAGTATAGTACAT

TACTAATTTTGTGGACTTCTTAATATTGCTTTAATCTTGAGTTATAGGAACTAAATTAAT

AAGTAGTTTTATCCTATCATTAAATTGCTCTAAATATGAATCTTAAAGACTTGAATGAGT

TTAAATATAATTCTAAAACTTATGGTAGAGCAATATAATGATTTCTTCTTCTCAAAGAAG

TAATGGAATCTATCAAAATTAAAACAGTAATGTAAAATCTTGGTCGAATTTCAGATCACT

GTATCATATATATGTAAATTGGACAAAGATAACACTGCCAAGCATGAAGTCTATGGTCTT
TTTTTTTTTTTTTTTTTTTTTTTGAGACAGGGTCTCGCTCTGTCACCCAGGCTGGAGTGC
AGTGGAGCGATCTCGGCTCACTGCAACCTCCATCTCCCAGGTTCAAGTGGTCCTCTCACC
TCAGCCTCCCGAGTAGCTGGGACCACAGGCACACACCACCACATCCAGCTATTATTTTGT
ATTTTTAGTAGATATGGGGTCTCATCATGTTGCCCAGGCTGATCTCAAACTCCTGAGCTC
AAGCGATCCGCTCACCTCGGCCTCCCAAAGTGCTGGGATTACAGGTGTGAGCCATCACGT
CCAGGCCCGATGGTCATTTTTAACCATAAT**T**CAATACCATGTGACAACTCAACTGCTATG
TACTAGTTGAGAATAAACAGTGTATGAATTATCTTCACCAATGGAATATCTATTCAAAAT
CAATAATAAGGGTTTCTTTTTTATTATTTCTAGTTTCTTATTTCTCTTTTTTTTTCTTGT
TTCAG**GGCTTTGAAGACTGCTTGGTATTTGATGAGTTAATGGATAAATTCAGTAACGACC**
TTAGTAAGTAAGGTCAATTTCTCAACTGGACATGTACTAGCTCTTCTGACTAATTGTGGG
CTTATTTCTGATCCTCAATGATCACAAACCTGTGATGTATAATGTATATTATCTCCCCAG
GATTACATATTTTTCTACTAATAGTCTATGCCACCGTTTTCCCATAGCAACAACTAACAC
CCAGAAAAAGAAACATAGTTTCTTTTGAAGTTTCTATGTGGAGGTCCATGAAGTGATTCA
CTCAGAGATTAAAGCTCAGAGTACCCGTGCTTGGCAGAAATAGGCTGCTTTCTCTCCCTT
TTCAGCCTAGGTAACACCCTCTGCCTGCGCCTCCTAGCAACAGTCCCAACGTTTTGGCAC
CAGGGACGGGTTTCACGGAAGACAGTGTTTCCACAGACAGGGGGTGGGGGGAGATGGTTT
CAGGATGAAACTGTTCCTGCTCTGATCTTCAGGCGTTAGTTGGATTCTCATAAGGAGCGT
GCAAGCTAGATGTCTCGCCTGTGCAGTTCACAATAGGGTTCACGCTCCTATGAGAAACTA
ATGCCACTGCTGATCTGACAGGAGGCGGAGCTTGGGCGGTAATGCCATCTCAACTGCCAC
TCACATCCTGCTGTGCGGCCCAGTTCCTAACAGGCCATGGACCGAGGGTTGGGGACAGTC
ACCCTACAACACATCCTCAGAAACACTTAGAAGCTCCAGTGTTTTTTGCCCTATCAAAGA
TTGCATCTAATACTTTTTTTTTTGAGATGGAGTCACTCCATCGCCCAGGCTGAAGTTCAG
TGGCCTCAGTCTTGGCTCACTGCAACCTCCGCCTCCTCTCCTGCCTCAGCCTCCAGAGTA
GCTGGGATTACAGGCATACACCACCACACCCAGCTAATTTTTGTATTTTTAGTAGAGACG
GGGTTTCGTCATGTTGGCCAGGCTGGTCTCGAACTCCTGACCTCAAGTGATCCACCCACC
TCTGCCTCCCAAAGTGCTGGGATTATAGGCGTGAGCCACCATGCCCGGCATAAATTCCTT
TTTTTTTTTTCATTATTTTTCAGTTATCTCTGTCAAATACTGAGGAGCCAGAGGCATTTT
GTGGAGGGTTTGTATATTACATATATAAAAGAAGGCACAAACAACAGCCTAAGAACTGGAT
AACCTTTTCCTACTTCCCAGCATCACGTGCTTTCAAAGGCCACCTCTGATTTTACACTTG
TTTCAGGTAAAGGATAGACTAGAGAACTGGCCTTAAACTTACACCCAGTTTCAAGTCCTG
ACTTGCCAACTTATTAACTGTGTGATCTTGAGCAAATCACTTCATCTTTCTAAAGCCTCC
GTTCTTTTTCATCTATAAAGTGGGGATGATACTCTCAACTTCAGAGTTGTGAACACTGAA
TAATTTCTATAAAGTTTTAAAAAGAAAAAAAAAGCCTACCGTAAAGTAAGTTCATAGTAT
ATGTTTGCTGTGGTGTTTTAGAAAGTCCAAGAATACTGGTGCCTGGGTCTGAAGAGATCA
TAGAATTCCAGGACAAATTTTCTCATCTTTTTTTTTCTTTTTTTTTTTTTTTTTTTTTTT
GGTTATTTCCCAGTCATCTCTCTCAGATGCTCAGGAGCCAGAGATATTTTGTTTCCTCCT
TTCCTTGTGCTCGCGTGACTGGTTTACACATGACGTTGGAGGTTGATAGAGGGTTTATGC

TSS underlined

***KMO*-exon 11**

CGI (highlighted)

KMO-BP(italic)

ncRNA-exon 2

Underlined

ATAAGAACACACTGTCAACGCTGGTTTGGGGCACTAGAGAACAAAGATAAAGCAATAATA
TAGATTAATTTTGTTGTAATTTTTATTTAATCACAACAGGAAATGCTCTGTGTATTGTCA
ATAGTCATAGATTGTTGGTTATACAGAATGGTTATTTATAGTTTAAAAAAATTAGCGAGA
GCATTGGTAGTGAACGTACCATTTAATTTGACCTGTGTAGAGTTCAGCACATTTTTATAG
AAGGTTTTGGAAAGATGTATTATTTAACTTACAG**GTTTGTGTCTTCCTGTGTTCTCAAGA**
TTGAGAATCCCAGATGATCACGCGATTTCAGACCTATCCATGTACAATTACATAGAGGTG
AGTGAGAAGGTTTGGCTTTATTCCATGAGATGGTTCACTGATATTCTAACAAGTGTTCTT
TTCTTTTTCTTTGCTTCAGCCCCCATCTCATGCATGCTATATAAGAATACCTACAGATAA
GAAAACACACACACACATAGCATTCCTTAGACTTGGGTGGATTGTGACTCTGGCCAGTGG
CAATGACCAATACACATTATCTCTATCTACACTTTAAGGACGTTTAGGAGGTAACAAAAC
CTCTGATAATTCACATGCTTTTATCTTAGACATTTAGCAATATGTTACTTTAATGAAAAT
ACAACTTTCAGATGTGTCAACATATTTAGAAAATCTAAGAAATGTTGAATGGCCTTTTCA
CTCATAACATTATAACTTGCATATTTAGAGTAGCATTGATTATCCTTTGGAAACTTATGC
ACTAAAAAAAAATGCATAATACCAAAAAGAAATTCATGTTCAGGAAGATTAGTACTTTGA
AAAAAAAACATATTAATTCCATTTTGAACCAGTCACATGTAAAGCTCATGGTAAGTGATT
GTGATTATAGTTTTAAAATAATAAAATGAAAGGATTTACAAGAT**AAATTTAAAGCAGAGT**
GACCAGCTATTTGCCATCTCTCCTGAAGACAGAAAGAAACTGGACTTCAGCTTGAGCAAA

Overlapping ncRNA-exon 1 and *KMO*-exon 12

Start of ncRNA
