# Supplementary material for: Kynurenine 3-Monooxygenase Gene Associated With Nicotine Initiation and Addiction: Analysis of Novel Regulatory Features at 5′ and 3′-Regions
Source: Front Genet. 2018 Jun 13;9:198. doi: 10.3389/fgene.2018.00198 (PMC6008986; doi:10.3389/fgene.2018.00198)

Supplementary Material

Kynurenine 3-Monooxygenase Gene Associated with Nicotine Initiation and Addiction: Analysis of Novel Regulatory Features at 5' and 3'-Regions

Hassan A. Aziz[1], Abdel-Salam Gomaa Abdel-Salam[1*], Mohammed A. Ibrahim Al-Obaide[2], Hytham W. Alobydi[3], Saif Al-Humaish[3]

*** Correspondence:** Corresponding Author: Abdo@qu.edu.qa

Figure S2. The differential expression of *KMO* mRNA in various human organs. Expression values from Affymetrix chips relate to fluorescence intensity; the biogps.org uses gcrma (Wu et al., 2013; Wu et al., 2016). The data extrapolated from excel sheet downloaded from Biogps.org database, $p < 0.05$.

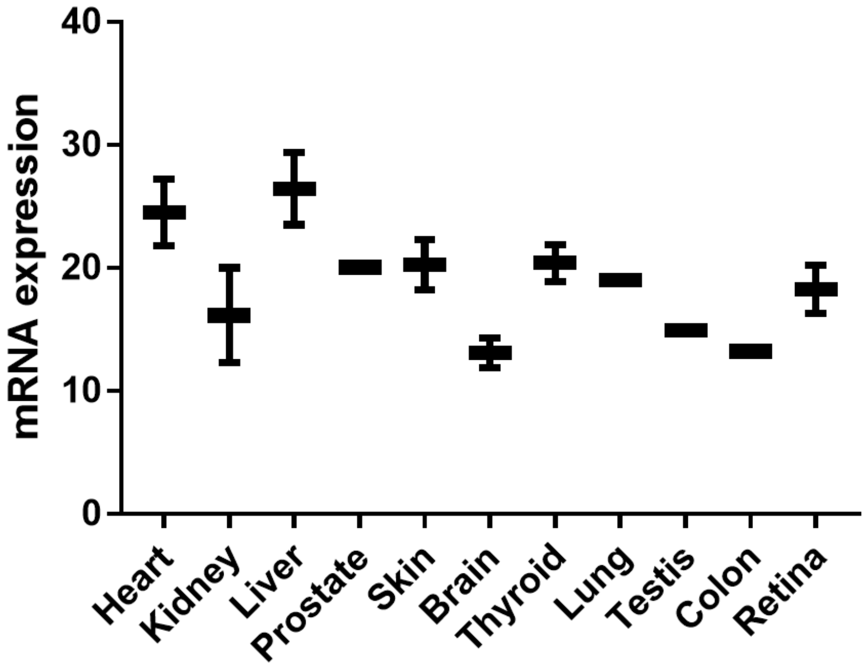

Supplement: Supplementary file 7 [file Data_Sheet_2.PDF]
