# Supplementary material for: Kynurenine 3-Monooxygenase Gene Associated With Nicotine Initiation and Addiction: Analysis of Novel Regulatory Features at 5′ and 3′-Regions
Source: Front Genet. 2018 Jun 13;9:198. doi: 10.3389/fgene.2018.00198 (PMC6008986; doi:10.3389/fgene.2018.00198)
Supplement: Supplementary file 8 [file Data_Sheet_3.PDF]

Supplementary Material

Kynurenine 3-Monooxygenase Gene Associated with Nicotine Initiation and Addiction: Analysis of Novel Regulatory Features at 5' and 3'-Regions

Hassan A. Aziz[1], Abdel-Salam Gomaa Abdel-Salam[1*], Mohammed A. Ibrahim Al-Obaide[2], Hytham W. Alobydi[3], Saif Al-Humaish[3]

*** Correspondence:** Corresponding Author: Abdo@qu.edu.qa

Figure S3. Alignment analysis by EMBOSS Matcher tool to show the similarity between the uncharacterized LOC105373233 ncRNA XR_949327.1 and *KMO* mRNA M_003679.4. We identified a sequence in the XR_949327.1 composed of 52 bps showed 75% similarity with the matching *KMO* NM_003679.4.

```
# Program: matcher
# Rundate: Wed 25 Apr 2018 21:26:49
# Commandline: matcher
```

sequence.

```
# Aligned_sequences: 2
# 1: NM_003679.4
# 2: XR_949327.1
# Matrix: EDNAFULL
# Gap_penalty: 16
# Extend_penalty: 4
#
# Length: 52
# Identity:      39/52 (75.0%)
# Similarity:    39/52 (75.0%)
# Gaps:           1/52 ( 1.9%)
# Score: 131
#
#
#=======================================

NM_003679.4     2419 CTATTTATTTA-TGTATTTAGAGATCAGGTCTCACTCTGTTGACCAGGCT   2467
                     ||||..|.||| |.||..||||||...|||.||||..|||||||||||||
XR_949327.1      368 CTATGAACTTACTTTACGTAGAGACGGGGTTTCACCATGTTGACCAGGCT    417

NM_003679.4     2468 GG   2469
                     ||
XR_949327.1      418 GG    419
```
